# Critical Comparison between Modified Monier-Williams and Electrochemical Methods to Determine Sulfite in Aqueous Solutions

**DOI:** 10.1100/2012/168148

**Published:** 2012-04-19

**Authors:** C. Montes, J. H. Vélez, G. Ramírez, M. Isaacs, R. Arce, M. J. Aguirre

**Affiliations:** ^1^Departamento de Química de Los Materiales, Facultad de Química y Biología, Universidad de Santiago de Chile (USACH), Avenida L.B. O'Higgins 3363, Estación Central, Santiago 9170022, Chile; ^2^Departamento de Química Inorgánica, Facultad de Química, Pontificia Universidad Católica de Chile, Vicuña Mackenna 4860, Santiago 7820536, Chile

## Abstract

In the present work, known concentration of sulfite aqueous solutions in the presence and absence of gallic acid was measured to corroborate the validity of modified Monier-Williams method. Free and bound-sulfite was estimated by differential pulse voltammetry. To our surprise, the modified Monier-Williams method (also known as aspiration method) showed to be very inaccurate for free-sulfite, although suitable for bound-sulfite determination. The differential pulse approach, using the standard addition method and a correction coefficient, proved to be swift, cheap, and very precise and accurate.

## 1. Introduction

Undoubtedly sulfur dioxide is the most widely spread additive in winemaking and essential as well. Antioxidant [1], antioxidasic [2], and antimicrobial [3, 4] effects turn sulfur dioxide into a practically essential additive not only in winemaking but also in other food production [5]. Sulfur dioxide may be found free or bound to phenols, for example, gallic acid, aldehydes, and other organic compounds [6].

On the other hand, a high level of this compound brings about toxic effects [7]. As its use is limited by regulation in every country, it is of the utmost importance to develop alternative methods that enable its rapid and cheap determination, for example, using electrochemical techniques [8–13].

Equilibrium among the different molecular species is reached when SO_2_ is added to wine. Part reacts with compounds having carbonyl groups and is called bound sulfite, the other part, that in aqueous solution is in equilibrium with bisulfite (HSO_3_^−^) and sulfur dioxide (SO_2_), is called free SO_2_ (1) [14, 15]. 

The concentration of these species will chiefly depend on pH. Under wine pH conditions, most free SO_2_ is present as bisulfite (HSO_3_^−^):

(1) $$\begin{array}{c} \;\text{S}{{\text{O}}_{3}}^{-2}+{\text{H}}^{+}⇌\text{HS}{{\text{O}}_{3}}^{-}\\ \text{HS}{{\text{O}}_{3}}^{-}+{\text{H}}^{+}⇌\text{S}{\text{O}}_{2}+{\text{H}}_{2}\text{O}. \end{array}$$

**Figure 1:**
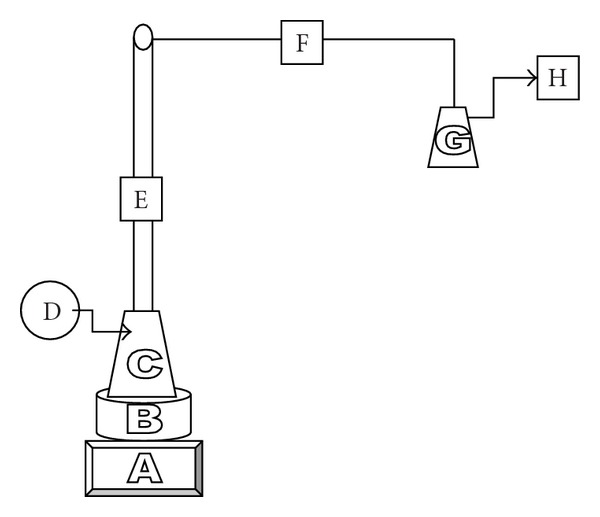
Arrangement for the determination of free and bound SO_2_ by the modified Monier-Williams method. (A) Heater, (B) glycerin bath, (C) flask 1, (D) air pump, (E) refrigerant, (F) connector, (G) flask 2, (H) air output.

Industry employs various methods for determining  SO_3_^−2^ or HSO_3_^−^ that are widely used in winemaking, for example, the modified method of optimized Monier-Williams method (2000) [16] (also known as aspiration method) (see Figure 1). Acidification of the sample is the key of the method [17] where the formed SO_2_ is drawn out by a nitrogen stream. The stream is then reacted with hydrogen peroxide to produce sulfuric acid (2) that is finally titrated with a 0.01 N NaOH standard solution. In the current research, this approach is utilized as control method. The procedure eliminates the interferences of pigments and acetic acid:

(2) $$\text{S}{\text{O}}_{2}+{\text{H}}_{2}{\text{O}}_{2}\to \text{S}{{\text{O}}_{3}}^{-2}+{\text{H}}_{2}\text{O}\to {\text{H}}_{2}\text{S}{\text{O}}_{4}.$$

Differential pulse voltammetry utilizing the standard addition was the selected electrochemical approach that will be compared to the aspiration method. The standard addition method [18, 19] is particularly useful for analyzing complex samples where interference due to the matrix (in real cases) is expected.

## 2. Experimental

### 2.1. Chemical Reagents

Na_2_SO_3_ (Merck, p.a.), NaCl (Merck, p.a.), C_6_H_2_(OH)_3_COOH (J.T. Baker), H_2_O_2_ (Vetc, 30%, p.a.), HCl and (Riedel deHaen, 37% p.a) (methyl red indicator Sigma-Aldrich, ultrapure N_2_ (AGA) were used as received.

### 2.2. Solutions

Fresh Na_2_SO_3_ solutions of different concentrations were prepared employing bidistilled and deionized water. NaCl about 100 times more concentrated was used as supporting electrolyte. 1 mM C_6_H_2_(OH)_3_COOH, 0.01 M NaOH (diluting 0.1 M standard solution), 0.3% (w/v) H_2_O_2_, and 25% (v/v) HCl solutions were also prepared.

### 2.3. Methods

**Modified Monier-Williams Method**

**Determination of Free and Bound Sulfite According to Modified Monier-Williams Method**

Free SO_2_ determination by the aspiration method was conducted as follows. 1 mM sodium sulfite solutions were prepared by weighing and dilution. Ten 20 mL aliquots of each solution are taken for each set of measurements and solutions were prepared in triplicate. For each measurement, 20 mL of sample were transferred into flask 1 and then 10 mL hydrochloric acid 25% v/v were added, and finally the flask was connected to the distillation setup. 10 mL 0.3% hydrogen peroxide and 5 drops of methyl red indicatorwere placed into flask 2. Free SO_2_ formed in flask 1 was removed by a stream of nitrogen or air at 1 L min^−1^ rate for 20 minutes (a 3 L min^−1^ gas flow afforded less accurate results). Besides, 30 minutes flow time produced identical results than flushing for 20 minutes). Then, sulfuric acid formed in flask 2 was titrated with 0.01 M NaOH solution. The end-point is taken by a color change from violet-blue to olive-green. Results obtained using nitrogen or air was identical and consequently all the measurements described here were obtained using air (see Figure 1).

Sulfite concentration was worked out using the equation reported in the literature [20]:

(3) $$\text{mg}\;{\text{L}}^{-1}\;\text{S}{\text{O}}_{2}=\frac{n\times N_{\text{NaOH}}\times 32\times 1000}{Vs},$$

where *n*: NaOH volume used in the titration and *Vs*: sample volume.

1 mM sodium sulfite and gallic acid solutions were used for total sulfite determination, and the temperature was kept at 85°C throughout the measurement using a glycerin bath. The difference between total and free sulfite corresponds to bound sulfite:

(4) $${\text{SO}}_{2}\;\left(\text{total}\right)={\text{SO}}_{2}\;\left(\text{bound}\right)+{\text{SO}}_{2}\;\left(\text{free}\right).$$

**Cyclic Voltammetry and Differential Pulse Voltammetry**

A three-compartment cell was utilized. Glassy carbon (geometric area 8.56 × 10^−4^ cm^2^) was used as working electrode, and a platinum coil of large area was the counter electrode. All potentials quoted in the current work are referred to an Ag/AgCl (3 M KCl) electrode provided with a Luggin capillary tip. Prior to each measurement, the working electrode was washed with distilled water and polished with alumina slurry. The working solution was previously deaerated by flushing with high-purity nitrogen for 10 minutes. A 1 M NaCl solution, to which NaOH or HCl was added to adjust pH, was employed as supporting electrolyte.

**Bound-Sulfite Determination by Differential Pulse Voltammetry Using Standard Addition**

**Figure 2:**
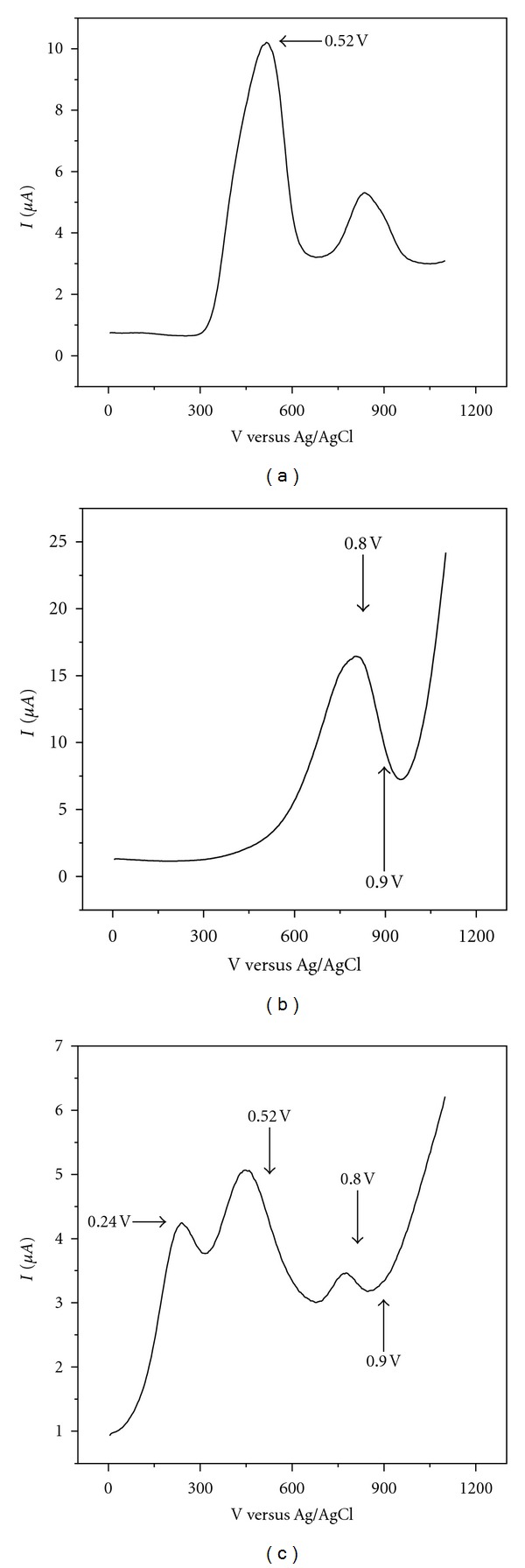
Cyclic voltammetry of (a) 10 mM SO_3_^−2^ solution at pH 9.5. (b) 1 mM gallic acid solution and (c) a gallic acid-sodium sulfite mixture. Initial concentration 10 mM, pH 9.5. Scan rate 0.1 mVs^−1^.

A cyclic voltammetry study was performed of solutions containing electrolyte and sulfite, electrolyte and gallic acid, and, also, 10 mM sodium sulfite +1 mM gallic acid solutions at pH 9.5. The obtained voltammograms are shown in Figure 2.

Results permit determining the potential at which the current must be measured for obtaining reproducible response related to sulfite concentration.

Measurements were accomplished by mixing 10 mL 1 mM gallic acid and 10 mL 2 mM sodium sulfite solutions. To this solution, ten 2 mL standard additions of sodium sulfite were performed and the respective voltammograms recorded. With the obtained data, sulfite concentration in the sample was calculated. The same procedure was performed for several samples to obtain a set of data. No HCl was added in this case.

## 3. Results

Free sulfite determined by the Monier-Williams modified method is listed in Table 1 (for a set of data).

These results were very reproducible and showed that for a 64 ppm solution, values *ca*. 43 ppm were obtained, very much lower than expected. Thus, the method showed high precision but poor accuracy. To determine if the accuracy problem was due to a too low air flow, unable to remove all SO_2_, this was increased three times. The obtained values were similar to those in Table 1 but less precise. Besides, the flow time was increased twice as much but no accuracy improvement was observed. On the other hand, it was assumed that the low accuracy could be a temperature problem, because total sulfite measured in the presence of gallic acid at 85°C showed good accuracy. Consequently, the experiments were repeated at 85°C. The found results were close to those illustrated in Table 1 but accompanied by a precision loss. These results are surprising since the described method appears in the literature as a very accurate one for free-sulfite determination [16]. Measurements were also accomplished using nitrogen instead of air, but the problem remained the same. Therefore, it was decided to apply a correction factor to the measurements generating thus Table 1 third column. Finally, the low accuracy was ascribed to a likely factor related to SO_2_ loss that would be generated in flask 1, during the acidification step, before the connection to the distillation arrangement was accomplished. This problem would not arise with bound sulfite owing to adduct formation that would stabilize sulfite at room temperature. In the winemaking business, this drawback would not exist because sulfite is always in contact with adduct-forming substances with various degree of stability at room temperature. However, it is a factor to be considered for sulfite determination in samples containing no “sulfite-ligands.” In such a case, the system design should be modified by adding a third mouth to flask 1 for in situ acidification with the air flow already circulating.

Applying 1.48 as correction factor, Table 1 statistical analysis [18] produced the following data:

(5) $$\begin{array}{c} \overset{®}{x}=64.02\;\text{ppm}\;\text{free}\;{\text{SO}}_{2},\\ S=1.98,\\ \text{CV}=3.1\text{\%},\\ \mu =64.02\pm 1.33. \end{array}$$

The method became accurate and reproducible by using a previously constructed calibration curve to determine the correction factor.

Total sulfite determination results using the modified Monier-Williams method are included in Table 2.

Statistical analysis of Table 2 afforded the following data:

(6) $$\begin{array}{c} \overset{®}{x}=63.05\;\text{ppm}\;\text{total}\;{\text{SO}}_{2},\\ S=0.96,\\ \text{CV}=1.53\text{\%},\\ \mu =63.05\pm 0.68. \end{array}$$

It can be clearly seen that the method works well with good accuracy and precision. A correction factor was not necessary in this case.

It seems that, as will be seen later, adduct formation between sulfite and gallic acid avoids losses by shifting equilibrium 1 to the left, preventing thus SO_2_ evaporation before the measurement starts.

As for electrochemical analysis, the voltammetric results (Figure 2) suggested that the signal appearing at 0.25 V for the gallic acid-sulfite mixture points to the formation of a new species, since this peak was not observed in the voltammograms of each separate analyte. The signal at 0.55 V would correspond to the first gallic acid oxidation, and the signal at 0.8 V would be mainly due to sulfite. This assignment was inferred by simple comparison of the three voltammograms.

**Figure 3:**
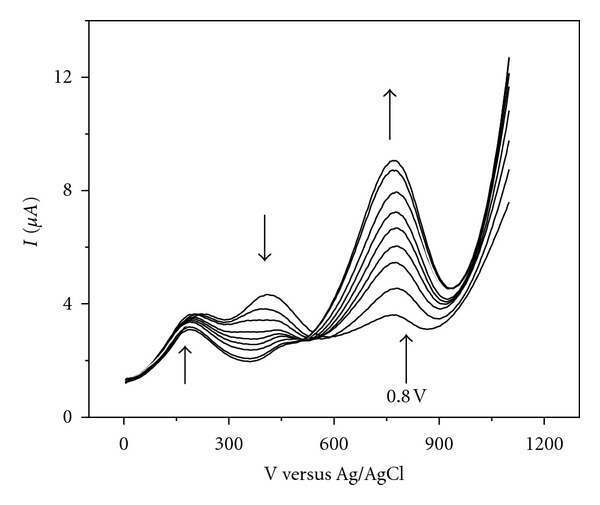
Differential pulse voltammetry profile of a gallic acid-sulfite mixture using standard addition method (ten 2-mL sulfite solution additions). Sulfite and gallic acid initial concentration 2 mM and 1 mM, respectively.

Quantitation employing the standard addition method was achieved by measuring the current at two potentials, namely, 0.8 and 0.9 V. The later one yielded the best results.  Figure 3 shows the obtained differential pulse voltammograms.

Results obtained by this technique with current measured at 0.9 V are presented in Table 3. Each result corresponds to a set of 10 measurements of a 2 mM sodium sulfite solution using the standard addition approach.

Statistical analysis of Table 3 yielded the following data:

(7) $$\begin{array}{c} \overset{®}{x}=2.00\times 10^{-3}\;\text{M},\\ S=1.2\times 10^{-4},\\ \text{CV}=10.5\text{\%},\\ \mu =2.00\pm 1.12\times 10^{-3}. \end{array}$$

The method proved to be appropriate, accurate, and reproducible. Similar results were obtained for free-sulfite utilizing the same method (not shown). In both cases, it is mandatory to construct a calibration curve to determine the correction factor, which is usually close to 1.

**Figure 4:**
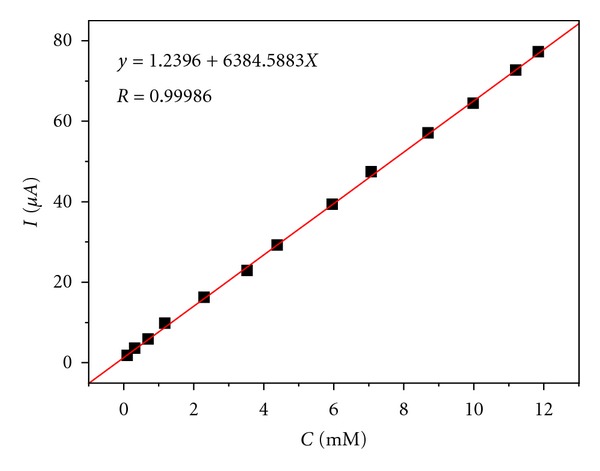
Calibration curve of sulfur determined by differential pulse voltammetry using standard addition.

Finally, the method was tested by varying sulfite concentration in the range usually employed in wines [20]. Excellent linear regression and detection limit low enough to become a suitable method for wines (see Figure 4) were obtained. From this figure, a linear range between 10 and 40 mg of sulfur per liter and a detection limit 7.2 mg L^−1^ can be inferred.

It is noteworthy that the obtained linear range can be extended to higher concentrations (not reported in the current work because they are outside the limits of sulfite found in wines). Compared to other methods (Table 4), although the reported here is not the best regarding to detection limit, it is very interesting indeed because of its low cost and because it requires neither sophisticated equipment nor modified electrodes, being very easy to implement on an industrial scale.

## 4. Conclusions

Differential pulse voltammetry using standard addition is a reproducible approach to determine sulfite in the presence and absence of gallic acid by measuring the current at 0.9 V. Previously, a calibration curve must be drawn to quantify the correction factor to be applied to current values.

The modified Monier-Williams method is suitable for the determination of total sulfite when working in the presence of gallic acid.

In the case of free-sulfite in samples that contain no substances that strongly interact with sulfite or bisulfite, it is necessary to modify flask 1 design and to acidify in situ.

## Figures and Tables

**Table 1:** Free SO_2_ concentration determined by the Monier-Williams modified method using 1 L min^−1^ air flow.

Sample no.	Free SO_2_ (ppm)	Free SO_2_ applying correction factor* (ppm)
1	42,43	62,79
2	43,59	64,52
3	45,91	67,94
4	43,18	63,91
5	42,84	63,41
6	45,10	66,75
7	45,91	60,56
8	42,92	63,52
9	42,34	62,67
10	43,11	63,81
11	43,46	64,32

*Correction factor: 1,48.

**Table 2:** Total SO_2_ concentration obtained in gallic acid-sulfite solutions by the modified Monier-Williams method.

Sample no.	Total SO_2_ Ppm
1	63,63
2	63,90
3	60,82
4	63,63
5	62,08
6	63,90
7	63,13
8	63,13
9	63,90
10	62,32

**Table 3:** Na_2_SO_3_ determination by DPV.

Sample no.	Na_2_SO_3_ (M)
1	1.97 × 10^−3^
2	2.13 × 10^−3^
3	1.85 × 10^−3^
4	2.07 × 10^−3^
5	1.98 × 10^−3^
6	2.14 × 10^−3^
7	1.85 × 10^−3^

Correction factor: 1.09.

**Table 4:** 

Technique	LOD	Concentration range	Method	Reference
DPV	0.3 uM	0.6–100 *μ*M	Oxidation of sulfite by ferrocenedicarboxylic acid modified multiwall carbon nanotube paste electrode	[10]
Flow injection analysis (FIA)	0.4 mg/L	0.5−50 mg L^−1^	Pervaporation-Flow injection with amperometric detection (Cooper hexacyanoferrate-carbon nanotube modified carbon paste electrode)	[12]
Amperometry	1.58 mg/L 47.5 *μ*M	3.85–33.8 mg L^−1^	Reduction of bisulfite by iron aminopolypyridyl conducting-glassy carbon modified electrode	[11]
Cyclic voltammetry	1.26 mg/L	0.4−70 mg L^−1^	Reduction of SO_2_ by porphyrin-nafion/composite modified glassy carbon electrode	[13]
DVP using standard addition	7.2 mg/L	10−40 mg L^−1^	Oxidation of sulfite at glassy carbon electrodes	This work
